# Sequential use of bronchial aspirates, biopsies and washings in the preoperative management of lung cancers

**DOI:** 10.1186/1742-6413-4-11

**Published:** 2007-06-04

**Authors:** Eric Piaton, Djamal Djelid, Bernard Duvert, Marielle Perrichon, Bernard Saugier

**Affiliations:** 1 Centre de Pathologie Est, Hôpital Femme-Mère-Enfant, 59, boulevard Pinel, 69677 Bron Cedex, France; 2 Université de Lyon, Université Claude Bernard Lyon 1, Lyon, France; 3 Services de Médecine Interne et Pneumologie 2 et 5, Centre Hospitalier Général de Lons-Le-Saunier, BP 364, 39016 Lons-le-Saunier, France

## Abstract

**Background:**

The combination of cytology and biopsies improves the recognition and typing of small cell (SCLC) versus non small cell (NSCLC) lung cancers in the fiberoptic bronchoscopy assessment of centrally located tumours.

**Methods:**

We studied whether bronchial aspirates performed before biopsies (BA) and washings performed after biopsies (BW) could increase the diagnostic yield of fiberoptic bronchoscopy. A series of 334 consecutive samples taken in patients with suspicious fiberoptic bronchoscopy findings was studied. Two hundred primary tumours were included in the study. The actual diagnosis was based on surgical tissue specimen analysis and/or imaging techniques. The typing used was that of the 1999 WHO/IASLC classification.

**Results:**

The diagnosis of malignancy and tumour typing were analyzed according to the sequential (combined) or single use of tests. Malignancy was assessed by cytology in 144/164 (87.8%) positive biopsy cases and in 174/200 tumour cases (87.0%). BA before biopsies allowed 84.0% of cancers to be diagnosed, whereas BW after biopsies allowed 79.0% of cancers to be found (p = ns). However, combining biopsies with BW allowed 94.0% of cancers to be diagnosed, whereas 82.0% were diagnosed by biopsies alone (p < 0.001). The highest diagnostic yield was obtained with the combination of BA, biopsies and BW, with 97.0% sensitivity. Exact concordance in typing was obtained in 83.8% of cases. The six surgically resected cases (3.0%) with negative cytology and biopsy results included four squamous cell carcinomas with necrotizing or fibrous surface and two adenocarcinomas, pT1 stage.

**Conclusion:**

Fiberoptic bronchoscopy may reach a yield of close to 100% in the diagnosis and typing of centrally located, primary lung cancers by combining bronchial aspirates, biopsies and washings.

## Background

Lung cancer is the most common cause of cancer worldwide, affecting about 200,000 men and women annually in the United States and in Europe. Cigarette smoking has been identified as the major risk factor of this type of cancer: 87 to 90% of all tracheal, bronchial and lung cancers may be attributed to smoking [1]. Even at present, the mortality rate of lung cancer remains over 85% at 5 years [1,2]. In France, the incidence rose from 47.4 to 52.2 per 100,000 in men and from 3.7 to 8.6 per 10,000 in women from 1980 to 2000 [3].

At time of diagnosis, about 90% of patients have symptoms related to local bronchopulmonary involvement, invasion of adjacent structures or metastases, general effects or paraneoplastic syndromes. After a chest radiography is performed, fiberoptic bronchoscopy is the most frequently used test [4], allowing the endobronchial lesions to be mapped and sampled.

Appropriate treatment of lung tumours usually means that the diagnosis is substantiated by biopsies and/or brushing and washing specimens [1]. Though histopathology remains the cornerstone of diagnosis, it is now widely accepted that cytology may provide critical information on the accurate typing of small cell (SCLC) versus non small cell lung cancers (NSCLC) and that it may have prognostic and therapeutic implications [5-8].

Bronchial washings, brushings and fine-needle aspirations complement biopsies in the diagnosis of lung cancer, with overall sensitivity of 88% for centrally located, endobronchial disease [9-11]. However the reliability of cytology in the diagnosis of lung cancer is difficult to assess, particularly when bronchial secretions are aspirated. Bronchial aspirates and washings are often considered as a whole, with sensitivity values ranging between 50% and 90% [1]. Washings and brushings have an 80% overall sensitivity but a low subtyping accuracy, especially in the NSCLC components [12].

We have previously shown that provided specific requirements are followed, patients with centrally located lung cancers are more likely to have positive cytology results than positive biopsy results [7]. The data we obtained were substantiated by fiberoptic bronchoscopy aspiration of secretions, a type of material which is often discarded because it is considered as yielding mainly degenerate debris, mucus and inflammatory background [1,10].

To our knowledge, these results have not been duplicated. One of the reasons is that diagnostic criteria, though well-known in other types of samples, are difficult to analyze in this kind of specimen: five slides per case are prepared (56 cm^2 ^screening area) and several tens of thousands of cylindric cells, metaplastic elements and macrophages are encountered [13]. Accordingly, the proportion of diagnostic cells may be very low and accurate diagnosis may only be achieved by a screening procedure associating cytotechnologists and cytopathologists.

A second step in our studies was to verify if sampling performed before (BA) and after (BW) mucosal biopsies could, as in other organs (brushings before and after biopsy in the endoscopic diagnosis of gastroesophageal malignancy) [14] allow better results to be obtained in the search for cancer cells. We therefore studied whether a combined approach could increase the diagnostic yield of fiberoptic bronchoscopy in a series of 200 consecutive, centrally located lung tumours.

## Methods

### Inclusion of cases

The study was approved by a local ethics committee. Over a 7-year period, 855 patients underwent fiberoptic bronchoscopy in the Departments of Internal Medicine and Pulmonology of Lons-le-Saunier General Hospital, France. Patients were referred by general practitioners because of prolonged cough, chest pain, dyspnea or acute symptoms of infection, or because of a haemoptysis episode. Patients had effusions or signs which could suggest an invasion of adjacent structures or metastases in 127 cases (14.8%).

All patients were examined and questioned about their smoking history, respiratory illnesses and symptoms, and possible environmental or occupational exposure to carcinogens. All had chest radiography in posteroanterior and lateral projections which were interpreted by a certified radiologist and by at least one pulmonologist.

Chest X-rays that showed no evidence of cancer were recorded as "negative", even when other non cancerous conditions (lobar emphysema, large bullae, diffuse emphysema, benign patterns of calcification) may have been present. Conversely, radiographic features such as atelectasis, focal increased lung density, recurrent or poorly resolving pneumonia associated or not with pleural reaction were recorded as suspicious.

Fiberoptic bronchoscopy was performed almost equally by four pulmonologists (BD, DD, MP and BS). As summarized in previous studies [9], the localisation and type of mucosal abnormalities were separated into six categories: 1) tumor growth (vegetation or endobronchial mass), 2) infiltration of the bronchial mucosa, 3) compression, extrinsic or intrinsic, 4) necrosis, 5) non-specific findings (localized redness of the mucosa, without swelling and irregularity), and 6) normal appearance.

After evaluation of both radiography and fiberoptic bronchoscopy, only centrally located lesions (e.g. corresponding to trachea and main bronchi) were selected for analysis. Among the 855 patients, 557 (65.1%) had negative fiberoptic bronchoscopy, negative cytology and no biopsy at first examination. 470 patients were not reviewed. The 87 remaining patients (15.6%) were seen at a later time during the 4-year period. All of them experienced a new fiberoptic bronchoscopy examination. Among them, 36/87 (41.4%) had suspicious chest X-ray and positive fiberoptic bronchoscopy, and were included in the series. Globally, 334 comparisons between cytology and biopsy were obtained in centrally located bronchial lesions, including 200 cancer cases.

### Biopsy specimens

Biopsies were taken from abnormal areas of the bronchial mucosa. Tissue specimens were fixed in Bouin's solution, processed routinely for paraffin embedding and stained with haematoxylin-eosin.

Examinations were performed by certified pathologists using the 1999 WHO/IASLC classification [15]. Only cases with unequivocal malignant features allowing tumour typing were considered as positive.

### Cytology specimens

Aspirates (BA) were obtained from the bronchial tree during fiberoptic bronchoscopy before biopsies (mean volume = 30 ± 15,8 ml). Secretions were directly aspirated through a flexible catheter inserted down the fiberoptic bronchoscope. After biopsies have been performed, a mean volume of 50 ml balanced saline solution was instilled and re-aspirated (BW) in order to rince the abnormal area.

Specimens were adequately separated and marked before dispatch. The samples obtained were fixed in 50% ethanol supplemented with 20 g/L polyglycol, PEG 1500 (Merck^® ^KgaA, VWR International, ref. 815005) and sent to the cytopathology laboratory. Upon receipt, specimens were centrifuged at 1500 RPM for 10 minutes. After the supernatant has been discarded, the cell pellet was aspirated and smeared on five Superfrost^® ^Plus slides (Menzel-Gläser, Braunschweig, Germany) in order to allow sufficient diagnostic accuracy [13]. After spray fixation (Cell-Fixx^®^, Thermo Electron Corp. Waltham, MA), slides were allowed to dessicate at room temperature for one hour. Slides were then stained according to a hypochromic Papanicolaou procedure.

Slides were screened by two certified cytotechnicians who recognized and marked all cellular abnormalities on 4 of the 5 slides. A single cytopathologist (EP) reviewed the marked points and screened the last slide, thus allowing a thorough examination of each sample.

When recognized, malignant cells were typed at high magnification (x400) and recorded as follows: squamous-cell carcinoma-type cells; adenocarcinomatous cells; large cell carcinoma-type cells; SCLC-type cells; poorly differentiated carcinomatous cells, none other specified; other cyto-histologic type including sarcomas and lymphomas, if applicable. However, from a practical point of view, tumours were separated into SCLC and NSCLC cases (Tables 1 and 2).

**Table 1:** Cross classification of combined bronchial aspirates and washings vs. biopsy results in 334 patients, including 200 lung cancer cases (%)

Biopsy					
	Us	Neg.	NSCLC	SCLC	Total

Aspirates					
Neg.	-	140* (84.3)	16 (11.9)	4 (13.3)	160 (47.9)
NSCLC	4 (ns)	24** (14.5)	116 (86.6)	2 (6.7)	146 (43.7)
SCLC	-	2 (1.2)	2 (1.5)	24 (80.0)	28 (8.4)

Total	4 (1.2)	166 (49.7)	134 (40.1)	30 (9.0)	334 (100.0)

Us, unsatisfactory for evaluation; ns, not significant; Neg, negative; NSCLC, non small cell lung cancer; SCLC, small cell lung cancer

*including 6 cancer cases proven by CT scan findings, subsequently operated, and having squamous cell carcinoma with necrotizing or fibrous surface, pT1-2 stage (n = 4) or adenocarcinoma, pT1 stage (n = 2)

**including 4 squamous cell carcinomas in situ cases

**Table 2:** Differences between the three steps of the procedure in the 334 cases studied

	BA	Biopsies	BW	n	Op.
Identical results (n = 264)	Neg.	Neg.	Neg.	140	6^§^
	NSCLC	NSCLC	NSCLC	104	104
	SCLC	SCLC	SCLC	20	2
Globally concordant (n = 16)	Us	NSCLC	NSCLC	2	2
	Neg.	NSCLC	NSCLC	2	2
	Neg.	SCLC	SCLC	2	2
	NSCLC	NSCLC	Us	2	2
	NSCLC	NSCLC	Neg.	6	6
	SCLC	SCLC	Neg.	2	2
Discordant cases (n = 54)	Neg.	NSCLC	Neg.	16	16
	Neg.	SCLC	Neg.	4	-
	NSCLC	Us	Us	4	4
	NSCLC	Neg.	Neg.	2	2
	NSCLC	Neg.	NSCLC	22^¶^	22
	SCLC	Neg.	SCLC	2	2
	NSCLC	SCLC	NSCLC	2*	2
	SCLC	NSCLC	SCLC	2*	2

Total				334	178

BA, bronchial aspirates (before biopsies); BW, bronchial washings (after biopsies); Op., operated; Us, unsatisfactory for evaluation; Neg, negative; NSCLC, non small cell lung cancer; SCLC, small cell lung cancer

^§^cancer cases found by surgical biopsy and subsequently operated

^¶^including 4 squamous cell carcinomas in situ

*combined squamous cell/large cell and small-cell carcinomas were diagnosed on subsequent surgical specimens in 2 cases

Recent studies have shown that there is no significant difference in survival rates between large cell neuroendocrine carcinomas and SCLC when stratified by stage [15]. Accordingly, we did not perform specific search for neuroendocrine markers in cases of unequivocal NSCLC, large cell carcinomas or typical SCLC.

### Statistics

Data were coded and computerized. The Chi-square test (standard or on paired series, when appropriate) was used to compare categorical variables, and a probability level of 0.05 was regarded as significant.

## Results

There were 334 combined cytology and biopsy samples obtained in 288 men and 46 women (mean age = 65.0 ± 11.5 years). Cases were included until a total of 200 cases with lung cancer were found. Tumours were confirmed by subsequent surgical specimens (178 cases), or by a follow up combining repeated CT scans, chest radiography and clinical outcome when surgery was not possible (22 cases).

The patients included had abnormal chest X-rays in 314 cases. Some other, referred for hemoptysis had a normal radiography (20 cases) but were also examined by fiberoptic bronchoscopy, cytology and biopsies. The fiberoptic bronchoscopy findings were as follows: tumour growth in 238 cases (71.3%), infiltration in 73 cases (21.9%) and non-specific findings in 14 cases (4.2%). Fiberoptic bronchoscopy was normal in only 9 cases (2.6%).

Comparison of cytological and biopsic results, taking into account the cyto-histologic typing of tumors, is shown in Table 1. Apart from one unsatisfactory biopsy, exact concordance was obtained in 280 of 334 cases (83.8%). The diagnosis of malignancy was assessed by cytology in 144 of 164 positive biopsy cases (87.8%) and in 174 of 200 tumour cases (87.0%). The false-negative rates of cytology and biopsy used separately were 24.0% and 18.0% respectively.

Comparative sensitivity values of 1) BA before biopsies, 2) BW after biopsies, 3) biopsies alone, 4) combined aspirates and 5) combined aspirates and biopsies together are shown in Table 3. BA before biopsies allowed 84.0% of cancers to be diagnosed, whereas BW after biopsies allowed 79.0% of cancers to be found (χ^2^_ps _= 1.64; p = ns). However, combining biopsies to BW allowed 94.0% of cancers to be diagnosed, whereas 82.0% were diagnosed by biopsies alone (χ^2^_ps _= 16.02; p < 0.001).

**Table 3:** Results of bronchial aspirates (BA), biopsies and bronchial washings (BW) according to their combined or uncombined use in 200 consecutive lung cancer cases

	Us (%)	Negative (% FN)	Positive (% Se)
BA before biopsies	2 (1.0)	30 (15.0)	168 (84.0)
BW after biopsies	6 (3.0)	36 (18.0)	158 (79.0)
Haemorrhagic	5 (83.3)	23 (54.8)	127 (80.4)
Biopsies alone	4 (2.0)	32 (16.0)	164 (82.0)
Aspirates combined	-	48 (24.0)	152 (76.0)
BA + biopsies	-	16 (8.0)	184 (92.0)
Biopsies + BW	4 (2.0)	8 (4.0)	188 (94.0)
BA + biopsies + BW	-	6 (3.0)	194 (97.0)

Us, unsatisfactory for evaluation

FN: false negative cases

Se: diagnostic sensitivity

There was no significant difference in sensitivity between biopsies + BA and biopsies + BW. The highest diagnostic yield was obtained with the combination of BA, biopsies and BW, with 97.0% sensitivity. Considering that biopsies alone were positive in only 82.0% of cancer cases, there was a 13.0%, 18.0% and 15.0% increase in sensitivity when BA and BW were associated to biopsies, in comparison with 1) BA before biopsies, 2) BW after biopsies and 3) biopsies alone, respectively.

The six cancer cases (3.0%) with negative cytology and biopsy results included 4 squamous cell carcinomas with necrotizing or fibrous surface (fiberoptic bronchoscopy showed a typical tumour growth) and 2 adenocarcinomas, pT1 stage (fiberoptic bronchoscopy showed a mucosal infiltration in one case, and was subnormal in the other).

Including unsatisfactory specimens, there remained 54 cases (16.2%) in which slightly discordant results could be evidenced between cytology and biopsies (Table 2). In all cases except four, there was agreement in cyto-histologic typing. In two cases, a SCLC component was demonstrated by cytology, whereas biopsies showed a large cell carcinoma. These cases were considered as combined small-cell/large cell carcinomas. In the two other cases, a squamous cell carcinoma was evidenced by cytology, whereas the biopsies demonstrated a SCLC. All initial discordances (small cell/large cell and squamous cell/SCLC) were also evidenced by subsequent surgical specimens.

## Discussion

According to the successive WHO classifications, and from a therapeutic point of view, it is preferable to separate lung tumours into SCLC that require intensive chemotherapy and sometimes radiation therapy, and NSCLC that are preferably treated surgically. Tumours with combined SCLC/large cell carcinoma components are frequently encountered, particularly in large surgical specimens [6]. It has been shown by the Pathology Committee of the International Association for the Study of Lung Cancer (IASLC) that cytology combined with biopsies could improve the recognition of combined SCLC/large cell lung carcinomas [6].

NSCLC cases represent about 80% of overall lung cancer cases worldwide [1]. Cell typing may be difficult, particularly on cytological and small biopsy samples, and the criteria used are not always uniform.

For these reasons, it has been accepted that the terms SCLC and NSCLC be used to report both biopsy and cytology specimens in which the cell type can be identified, but clear-cut morphological criteria are lacking [8,16,17].

Exact concordance in typing may exceed 80%, regardless of the cytological method used (sputum, bronchial aspirates, washings with isotonic saline and brushings) [5,7,11]. In the present series, we evidenced two cases in which a SCLC component was diagnosed by cytology and a large cell carcinoma by biopsies.

Cytologic specimens collected from fiberoptic bronchoscopy yield a higher rate of positive results than sputum, particularly when considering washings and brushings. Direct aspirates without washing are generally not considered as a valuable diagnostic material. However, the sensitivity of secretions in diagnosing lung cancers has been reported to be 81.0%, with no falsely positive results, and can therefore match values obtained with washings [5].

In a previous study, we have demonstrated that an 88.3% sensitivity value can be obtained, if one considers initial biopsy results as the diagnostic reference, and 90.4% if both histopathology and follow-up (considered as the gold standard) are taken into account [7]. Data about bronchial aspirates remain rare, mainly because authors find it difficult to obtain representative material, thus leading them to favour other cytological methods [4,16]. As shown previously, washings and brushings have relatively high diagnostic yields (about 80%). However, neither adds significantly to the yield of biopsies [16].

Another limiting factor is the screening time of aspirates and washings, which must be smeared on five slides per specimen in order to reach a sufficient diagnostic sensitivity. Smearing accounts for a 56 cm^2 ^screening area for each specimen and a 15 to 45 min./sp. screening time, according to the difficulties encountered [7].

Fiberoptic bronchoscopy and biopsies allow about 90% of visible lung tumours to be diagnosed. However, biopsies cannot be performed in certain cases, particularly in ambulatory patients with a haemorrhagic risk and in patients with severe underlying diseases. Additionally, there are cases where malignancy cannot be proven, whereas fiberoptic bronchoscopy shows unquestionable tumour growth. In such cases, the tumour surface is soft and necrotic, or unusually hard and fibrous and may not provide histologically identifiable material [16].

In the present study, performing both examinations (BA, BW and biopsies) did not allow any unsatisfactory sample to be obtained, reduced the false-negative rate of cytology and significantly increased the diagnostic sensitivity of tests versus biopsies alone. Washings performed after biopsies (BW) were always found to be haemorrhagic. In our experience however, blood did not prevent us from recognizing and typing tumour cells.

Accordingly, we conclude that fiberoptic bronchoscopy may reach a yield of close to 100% in the diagnosis and typing of centrally located, primary lung cancers by combining bronchial aspirates, biopsies and washings.

## Abbreviations

Bronchial aspirate before biopsy (BA)

Bronchial washing after biopsy (BW)

Small cell lung cancer (SCLC)

Non small cell lung cancer (NSCLC)

## Competing interests

The author(s) declare that they have no competing interests.

## Authors' contributions

EP performed the data collection and the statistical analysis, performed the literature search and prepared the manuscript. DD, BD, MP and BS performed the clinical, radiological and fiberoptic bronchoscopy examinations, and they provided the follow up data.

**Figure 1:**
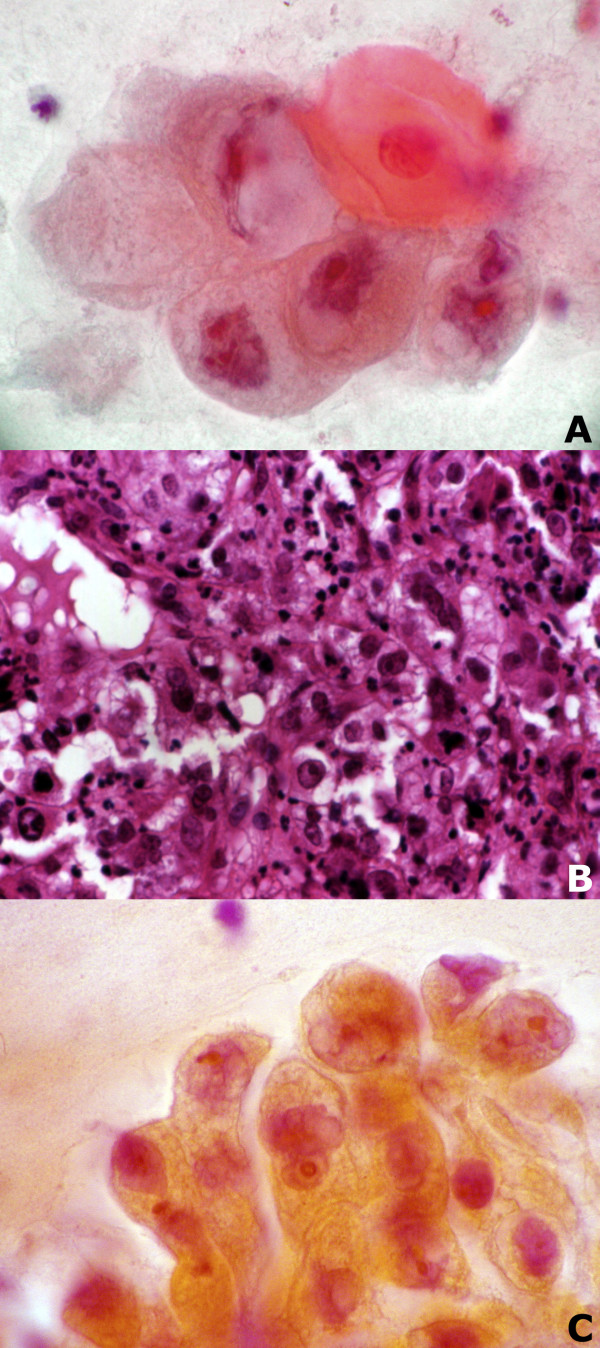
Bronchial aspirate before biopsy (A), biopsy specimen (B) and bronchial washing after biopsy (C) in a case of NSCLC, large cell carcinoma type (Papanicolaou stain, x630 (A and C), Haematoxylin and Eosin stain x 200 (B)).

**Table 4:** Cyto-histological correlations in 178 operated cases

Surgical specimen				
BA and BW	Squ.CC		LCC	SCLC

Neg.*	4	2	-	-
Squ.CC	31^¶^	7	8	-
AdenoC.	7	54	21	2
LCC	10	16	6	-
SCLC	2	1	1	6^§^

Total	54	80**	36	8

BA, bronchial aspirates (before biopsies); BW, bronchial washings (after biopsies); Neg, negative; Squ.CC, squamous cell carcinoma; AdenoC., adenocarcinoma; LCC, large cell carcinoma; SCLC, small cell lung cancer

*six cases with negative cytology and biopsies had positive surgical biopsy specimen and were subsequently operated

^¶^including 4 squamous cell carcinomas in situ

^§^some patients with T1-2 SCLC without sign of lymph node involvement underwent surgery followed by adjuvant chemotherapy

**37% of cases only showed a pure histological pattern
